# Glycyrrhizic acid alleviates bleomycin-induced pulmonary fibrosis in rats

**DOI:** 10.3389/fphar.2015.00215

**Published:** 2015-10-01

**Authors:** Lili Gao, Haiying Tang, Huanyu He, Jia Liu, Jingwei Mao, Hong Ji, Hongli Lin, Taihua Wu

**Affiliations:** ^1^Department of Respiratory Medicine, The First Affiliated Hospital of Dalian Medical University, DalianChina; ^2^Department of Gastroenterology, The First Affiliated Hospital of Dalian Medical University, DalianChina; ^3^Department of Pediatrics, The First Affiliated Hospital of Dalian Medical University, DalianChina; ^4^Department of Nephrology, The First Affiliated Hospital of Dalian Medical University, DalianChina

**Keywords:** glycyrrhizic acid, pulmonary fibrosis, bleomycin, transforming growth factor-beta, fibroproliferation

## Abstract

Idiopathic pulmonary fibrosis is a progressive and lethal form of interstitial lung disease that lacks effective therapies at present. Glycyrrhizic acid (GA), a natural compound extracted from a traditional Chinese herbal medicine *Glycyrrhiza glabra*, was recently reported to benefit lung injury and liver fibrosis in animal models, yet whether GA has a therapeutic effect on pulmonary fibrosis is unknown. In this study, we investigated the potential therapeutic effect of GA on pulmonary fibrosis in a rat model with bleomycin (BLM)-induced pulmonary fibrosis. The results indicated that GA treatment remarkably ameliorated BLM-induced pulmonary fibrosis and attenuated BLM-induced inflammation, oxidative stress, epithelial-mesenchymal transition, and activation of transforming growth factor-beta signaling pathway in the lungs. Further, we demonstrated that GA treatment inhibited proliferation of 3T6 fibroblast cells, induced cell cycle arrest and promoted apoptosis *in vitro*, implying that GA-mediated suppression of fibroproliferation may contribute to the anti-fibrotic effect against BLM-induced pulmonary fibrosis. In summary, our study suggests a therapeutic potential of GA in the treatment of pulmonary fibrosis.

## Introduction

Idiopathic pulmonary fibrosis (IPF) is a progressive, usually fatal, form of interstitial lung disease of unknown etiology. IPF is characterized by areas of peripheral fibrosis, interstitial inflammation, failure of alveolar re-epithelialization, persistence of fibroblasts/myofibroblasts, and deposition of extracellular matrix (ECM) molecules in the lung, and it resembles the histopathological pattern of usual interstitial pneumonitis (UIP; White et al., 2003). The progression of IPF may ultimately result in distortion of lung architecture and respiration failure, and the estimated 5-years survival of IPF is approximately 20% (King et al., 2001).

Fibroblasts play a critical role in the repair and regenerative process in almost all human tissues. Following injury, clusters of fibroblasts with an activated myofibroblast phenotype are transiently present in the granulation tissue, namely fibroblast foci, and they represent microscopic zones where fibroblasts migrate, proliferate, and secrete ECM proteins that provide a tissue scaffold for the repair process (Lorena et al., 2002). The activated fibroblasts, or myofibroblasts, are intermediates between fibroblasts and smooth muscle cells, simultaneously producing collagen and expressing α-smooth muscle actin (α-SMA; Hu and Phan, 2013), and they are involved in the physiological wound healing process and in the pathological fibrosis (Lorena et al., 2002; Kramann et al., 2013). Fibroblast foci have long been described in IPF (Katzenstein and Myers, 1998), and fibroblasts/myofibroblasts are recognized to be the key effector cells in fibrogenesis and in the pathogenesis of IPF (White et al., 2003).

The conventional therapeutic strategies for IPF with corticosteroids and immunomodulatory agents are based on the paradigm that chronic inflammation leads to prolonged tissue injury and fibrosis. However, they are largely ineffective and sometimes render prominent adverse effects (Flaherty et al., 2001; Spagnolo et al., 2015). Hence, searching for novel therapeutic agents with high efficacy and low toxicity is critical to manage disease progression and ultimately cure the patients with IPF.

Glycyrrhizic acid (GA), which is extracted from a traditional Chinese herbal medicine *Glycyrrhiza glabra*, is a major active component in *G. glabra* and has been demonstrated by modern scientific approaches to possess a wide spectrum of pharmaceutical properties, such as anti-inflammatory, anti-diabetic, anti-oxidant, anti-tumor, anti-microbial, and anti-viral properties (Ploeger et al., 2001; Ming and Yin, 2013). GA and derivatives have been shown to exert beneficial effects in the lungs against chemical-induced acute lung injury in animal models by modulating the expression and activity of several redox enzymes (Ni et al., 2011; Qamar et al., 2012). Moreover, GA can alleviate asthma in ovalbumin-sensitized mice via the immunoregulatory effects on T cells (Ma et al., 2013). Recently, GA was implicated to inhibit CCl_4_-induced liver fibrosis in rats (Guo et al., 2013; Liang et al., 2015), suggesting a protective effect of GA against progressive fibrosis after tissue injury. Yet, whether GA has a protective effect against pulmonary fibrosis remains unclear.

This study aimed to investigate the potential therapeutic effect of GA for IPF using a rat model with bleomycin (BLM)-induced pulmonary fibrosis. The results demonstrated that GA treatment significantly ameliorated BLM-induced pulmonary fibrosis, inflammation, oxidative stress, epithelial-mesenchymal transition (EMT) and activation of transforming growth factor-beta (TGF-β) signaling pathway in the lungs. Furthermore, the *in vitro* study revealed that GA could suppress proliferation, promote apoptosis and inhibit migration of fibroblast cells.

## Materials and Methods

### Establishment of BLM-induced Pulmonary Fibrosis in Rats and GA Treatment

Forty male 8-weeks-old Sprague-Dawley rats, weighing around 250 g, were used in this study. The rats were hosted in compliance with the international guidelines of laboratory animal care, and the procedures on animals were approved by the Institutional Animal Care and Use Committee of Dalian Medical University. The rats were randomly divided into five groups with eight rats in each group: control, BLM, BLM+GA_50_, BLM+GA_100_, and BLM+GA_200_. Induction of pulmonary fibrosis with BLM was conducted according to a previously described method (Thrall et al., 1979). All rats were anesthetized with 10% hydrate chloride (Sinopharm, Shanghai, China) at 3.5 ml/kg body weight (bw). Following anesthesia, a midline cut of the neck skin was made, and the trachea was exposed by blunt dissection. The needle of 1 ml syringe was inserted into the trachea, and bleomycin (Melonepharma, Dalian, China), dissolved in 100 μl sterile saline, was injected into the rat’s lungs at a dose of 5 mg/kg bw, while an equal volume of saline was injected into the rats from the Control group. The rats were rotated immediately after injection to ensure an even distribution of BLM in the lungs, and then the neck skin incision was sewn. Thereafter, the rats from the BLM+GA_50_, BLM+GA_100_, and BLM+GA_200_ groups received an intraperitoneal injection of GA at a dose of 50, 100, and 200 mg/kg bw respectively every day for a total of 28 days, and the rest animals received saline. The rats were sacrificed 28 days after BLM induction, the bronchoalveolar lavage fluids (BALFs) were collected by intratracheal instillation and draining of 1.5 ml saline for three times, and then the lungs were excised for further analysis.

### Histopathological Examination

The lungs were fixed, paraffin embedded, sectioned at 5 μm and stained with hematoxylin and eosine (H&E; Solarbio, Beijing, China) for microscopic examination of morphological changes. The sections were also subjected to Masson staining to identify collagen fibers. Briefly, the sections were incubated with hematoxylin for 6 min to stain the nuclei and then with Ponceau-Fuchsin acid solution (0.7% w/v ponceau, 0.3% w/v fuchsin acid, 1% v/v glacial acetic acid, all from Sinopharm, Shanghai, China) for 1 min to stain the cytoplasm. After washing with 0.2% glacial acetic acid, the sections were incubated with 1% phosphomolybdic acid for 5 min to destain the connective tissue, and the collagen fibers were stained by aniline blue (2% w/v aniline blue + 2% v/v glacial acetic acid) for 5 min. The sections were dehydrated, mounted, and observed at 200 × magnification.

### Lung Index Assay

A fraction of the lung was cut off and weighed prior to and following drying in an incubator at 60°C for 72 h. The pulmonary edema was calculated as the wet/dry (W/D) weight ratio.

### Determination of Collagen I and Hydroxyproline Levels in the Lung

To determine the content of collagen I in the lungs, exactly 50 mg lung tissue was physically homogenized in PBS and subjected to freezing-thaw in liquid nitrogen for three times. The level of collagen I in the tissue homogenate was determined with the Collagen Type I Alpha 2 ELISA (enzyme-linked immunosorbent assay) Kit (Cat. No.: SEA571Ra, USCN, Wuhan, China) following the manufacturer’s instructions. The tissue homogenates were centrifuged and the supernatant was collected. The protein concentration in the supernatant was determined with the BCA Assay Kit (Beyotime, Haimen, China), and 0.5 mg protein sample was used to measure the content of hydroxyproline using the Hydroxyproline Assay Kit (Cat. No.: A030-1, Jiancheng, Nanjing, China) according to the manufacturer’s instructions.

### Differential Cell Count in BALF

The BALF was centrifuged at 1,000 *g* for 10 min at 4°C, and the supernatant was stored immediately at -80°C until analysis. The cell pellet was resuspended in 0.5 ml PBS, and 10 μl of the cell suspension was made into the smear on a glass slide. The cell smear was air-dried, fixed in methanol for 15 min, and stained with Giemsa solution (Jiancheng). The total and differential leukocyte counts were determined under 400× magnification.

### BALF Biochemical Analysis

The levels of tumor necrosis factor-α (TNF-α), interleukin (IL)-1β, and IL-6 in BALF were determined by ELISA using the commercially available kits (Cat. No.: SEA133Ra, SEA563Ra, and SEA079Ra, USCN) in accordance with the manufacturer’s instructions.

### Determination of the Level of MDA and the Activity of MPO in the Lung

Lung tissue homogenates were subjected to the assay of myeloperoxidase (MPO) activity using the MPO Assay Kit (Cat. No.: A044, Jiancheng) according to the manufacturer’s instructions. The lung homogenates were centrifuged and the supernatant was collected. The protein concentration in the supernatant was determined with the BCA Assay Kit, and the level of malondialdehyde (MDA) was determined with the MDA Assay Kit (Cat. No.: A003-1, Jiancheng) following the manufacturer’s instructions.

### Immunofluorescence Staining

The lung sections were dewaxed, heated in the antigen retrieval reagent (18 mM citric acid and 82 mM sodium citrate) for 10 min and blocked with goat serum (Solarbio). The sections were incubated with anti-E-cadherin antibody (1:100, Cat. No.: BA0474, Boster, Wuhan, China) overnight at 4°C, followed by incubation with Cy3-conjugated goat anti-rabbit IgG antibody (1:200, Cat. No.: A0516, Beyotime) for 1 h at room temperature. Thereafter, the cell nuclei were briefly stained with DAPI (Biosharp, Korea). The sections were washed, mounted and observed under a BX53 fluorescence microscope (Olympus, Japan).

### Immunoblotting

For total protein extraction, lung tissues were physically homogenized and lysed with RIPA lysis buffer containing 1% v/v PMSF (Beyotime), and the cultured cells were lysed with NP-40 lysis buffer (Beyotime). Protein concentration was determined with the BCA Assay Kit. A total of 40 μg proteins from each sample were separated by SDS-PAGE, and then transferred onto PVDF membranes (Millipore, Bedford, MA, USA). The membranes were blocked with 5% non-fat milk and incubated with a specific primary antibody against the protein of interest overnight at 4°C. Anti-TGF-β1 (Cat. No.: sc-146), anti-Cyclin B1 (Cat. No.: sc-245), and anti-P53 (Cat. No.: sc-6243) antibodies were purchased from Santa Cruz (Dallas, TX, USA); anti-Smad2 (Cat. No.: bs-0718R), anti-p-Smad2 (Cat. No.: bs-5618R), anti-Smad3 (Cat. No.: bs-3484R), anti-p-Smad3 (Cat. No.: bs-5459R), and anti-Vimentin (Cat. No.: bs-8533R) antibodies were from Bioss (Beijing, China); anti-E-cadherin (Cat. No.: BA0474), anti-Fibronectin (Cat. No.: BA1772), anti-α-SMA (Cat. No.: BM0002), anti-Cyclin D1 (Cat. No.: BM0771), anti-Cyclin E (Cat. No.: BA0774), anti-P21 (Cat. No.: BA0272), anti-Bcl-2 (Cat. No.: BA0412), anti-MMP-3 (Cat. No.: BA1531), anti-MMP-7 (Cat. No.: PB0071), anti-MMP-8 (Cat. No.: BA2201), and anti-MMP-9 (Cat. No.: BA2202) antibodies were purchased from Boster; anti-cleaved caspase-3 (Cat. No.: ab2302) and anti-PARP (Cat. No.: ab32561) antibodies were from abcam (Cambridge, MA, USA); antibodies against cleaved caspase-8 (WL0153) and cleaved caspase-9 (WL01551) were purchased from Wanleibio (Shenyang, China). After incubation with the primary antibody, the membranes were incubated with horseradish peroxidase (HRP)-conjugated goat anti-mouse or goat anti-rabbit IgG secondary antibody (Beyotime) at room temperature for 45 min, followed by signal visualization using the enhanced chemiluminescence (ECL) system (7Sea Biotech, Shanghai, China). The membranes were stripped with the stripping buffer (Beyotime) and re-probed with anti-β-actin antibody (Cat. No.: sc-47778, Santa Cruz) to verify equal loading and transfer. The blot films were scanned and analyzed with Gel-Pro-Analyzer software to quantify the densitometric values of the target bands.

### Cell Culture and Treatment

Murine fibroblast cell line 3T6 was purchased from the Cell Bank of China Academy of Sciences (Shanghai, China). The cells were cultured in DMEM (Gibco, Carlsbad, CA, USA) supplemented with 10% FBS (Hyclone, Logan, UT, USA) at 37°C in a humidified atmosphere of 95% air and 5% CO_2_.

GA was dissolved in DMSO to make 180 mM concentrated stocks, which were diluted with PBS to make the 9 mM working solution. 3T6 cells were treated with 5, 10, 25, 50, 100, and 200 μM GA for 24 h, and the cytotoxicity was then analyzed by measuring the activity of lactate dehydrogenase (LDH) in the conditioned culture medium using the LDH Activity Assay Kit (Cat. No.: A020-2, Jiancheng). Low (25 μM), medium (50 μM), and high (100 μM) doses of GA were selected to treat the cells in later experiments.

### Proliferation Assay

Cell proliferation was assessed by the 3-(4,5-dimethylthiazol-2-yl)-2,5-diphenyltetrazolium bromide (MTT) assay. 3T6 cells were seeded in 96-well microplates at a density of 3,000 cells per well and cultured at 37°C for 24 h. GA was added into each well to the indicated final concentration (0, 25, 50, or 100 μM). After 24 or 48 h GA treatment, MTT (Sigma-Aldrich, St. Louis, MO, USA) was added into the culture medium to a final concentration of 0.2 mg/ml for 4 h incubation at 37°C. Thereafter, the medium was aspirated and the formazan crystals were dissolved completely in 200 μl DMSO per well. The optical density (OD) at 490 nm was recorded by an ELX-800 microplate reader (BioTek, Winooski, VT, USA). Each assay point was done in five replicates.

### Flow Cytometric Analysis of Cell Cycle and Apoptosis

Following GA treatment for 24 h, the cells were analyzed for cell cycle and apoptosis by flow cytometry. For cell cycle analysis, the cells were harvested, fixed in 70% ethanol at 4°C for 2 h, and incubated with the propidium iodide (PI) solution (Beyotime) for 30 min at 37°C in the dark, followed by analysis in FACSCalibur flow cytometer (BD Biosciences, Franklin Lakes, NJ, USA). Cell apoptosis was assayed with the Annexin V-FITC/PI Apoptosis Detection Kit (Cat. No.: KGA106, KeyGen, Nanjing, China) according to the manufacturer’s instructions. Following staining, the apoptotic status of the cells was analyzed by flow cytometry.

### Scratch Wound Assay

Cell migration was assessed by the well-established *in vitro* scratch wound assay (Liang et al., 2007). The confluent monolayer of 3T6 cells was incubated with 5 μM mitomycin-C (Sigma-aldrich) for 2 h to inhibit cell proliferation. A scratch was evenly created by horizontally crossing the surface of the cell monolayer with a 200 μl pipette tip. The detached cells were washed off with serum-free medium, and the cells were cultured with serum-free medium containing the indicated concentration of GA for 24 h at 37°C in a 5% CO_2_ incubator. The cells were photographed under an inverted microscope at 0, 6, 12, and 24 h post-scratching, and the rate of wound closure was calculated as (original gap distance - gap distance at the indicated time point)/original gap distance × 100%.

### Transwell Assay

3T6 cells were pre-treated with 5 μM mitomycin-C for 2 h, and resuspended in culture medium containing the indicated concentration of GA. 2 × 10^4^ cells in 200 μl suspension were plated in one Transwell chamber (Corning) pre-coated with Matrigel (BD Biosciences). The Transwell chamber was then placed into a 24-well plate with each well-containing 800 μl culture medium supplemented with 20% FBS. The cells were cultured for 24 h at 37°C in an atmosphere of 5% CO_2_. Thereafter, the cells and the Matrigel on the top surface of the Transwell membrane were wiped off, and the cells on the bottom surface of the membrane were fixed with paraformaldehyde and stained with crystal violet (Amresco, Solon, OH, USA). The cells were observed under a 200 × inverted microscope, and the numbers of the invading cells were counted in five fields on each membrane.

### Statistical Analysis

Data were processed with the GraphPad PRISM software (version 5.0; San Diego, CA, USA), and are presented as the mean ± standard deviation (SD). One way analysis of variance (ANOVA) was used to compare differences among multiple groups, followed by Bonferroni *post hoc* test for comparisons between two groups. The differences are considered statistically significant when *p* < 0.05.

## Results

### GA Attenuated BLM-induced Pulmonary Fibrosis in Rats

**FIGURE 1:**
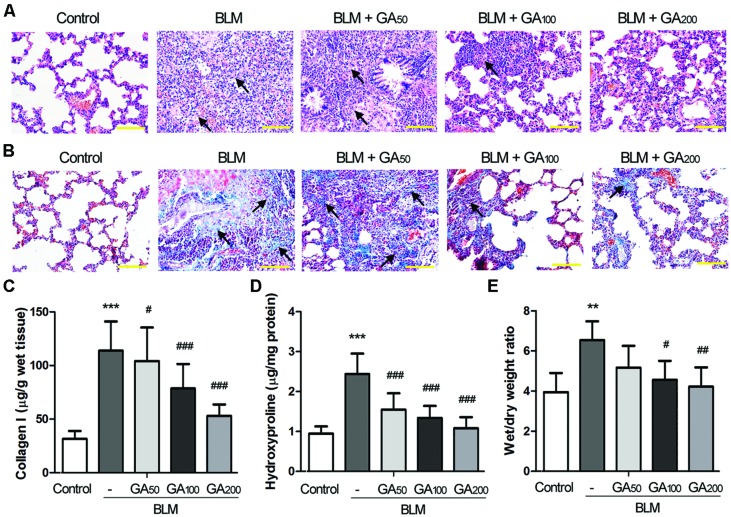
**Glycyrrhizic acid (GA) alleviated BLM-induced pulmonary fibrosis in rats.** Pulmonary fibrosis was induced by intratracheal instillation of BLM, and the rats received various doses of GA by intraperitoneal injection for 28 days (*n* = 8 per group). **(A)** Histomorphological examination (200 × magnification) of the lung sections following H&E staining. Scale bars = 100 μm. The arrows point at typical areas of fibrosis, manifesting as clusters of cells with protruding cytoplasm, i.e., the fibroblasts. **(B)** Masson’s staining of collagen (blue) in the lungs (200 × magnification). Scale bars = 100 μm. The arrows point at typical areas of fibrosis enriched in fibroblasts and collagen fibers. **(C,D)** The levels of collagen I and hydroxyproline in the lungs determined by ELISA. **(E)** Wet/dry weight ratio of the lungs. This figure shows the representative images from each group, and the results are expressed as the mean ± standard deviation. Compared with the control group, **p < 0.01, ***p < 0.001; compared with the BLM group, ^#^*p* < 0.05, ^##^*p* < 0.01,^###^*p* < 0.001.

Pulmonary fibrosis was primarily assessed by the histomorphological examination following H&E staining of the lung sections. Twenty-eight days after intratracheal instillation of BLM, marked thickening in alveolar septa, collapse of alveolar spaces, loss of alveolar structure, and over-proliferation of fibroblasts were observed in the lungs from the BLM group (**Figure 1A**), indicating a severe pulmonary fibrosis induced by BLM. GA treatment at a dose of 50 mg/kg bw/d (GA_50_) slightly alleviated BLM-induced pathological changes in the lungs, while higher doses of GA (GA_100_ and GA_200_) significantly ameliorated BLM-induced pulmonary fibrosis. Local fibrotic lesions were detected in the lungs from the BLM+GA_100_ group, and GA at a dose of 200 mg/kg bw/d (GA_200_) greatly maintained the alveolar structure after BLM administration with mild thickening of the lung interstitium. In addition to H&E staining, pulmonary fibrosis was verified by Masson’s staining of collagen. As shown in **Figure 1B**, the area of collagen deposition spread throughout the entire lung of the rats treated with BLM, whereas GA_100_ and GA_200_ remarkably reduced the areas of collagen deposition after BLM treatment. Moreover, collagen accumulation was quantified by measuring the contents of collagen I and hydroxyproline in the lung (**Figures 1C,D**). BLM treatment resulted in prominent elevation in the levels of collagen I and hydroxyproline in the lungs as compared with the control lungs (*p* < 0.001). Such elevation was significantly reduced by GA treatment in a dose-dependent manner (*p* < 0.05, *p* < 0.001). Since the thickening of the lung interstitum may also due to extra fluid (edema; Dongaonkar et al., 2009), the wet/dry weight ratio of the lung tissues was determined. The results revealed that BLM-induced pulmonary edema was alleviated by GA treatment, and the effect of GA was dose dependent (**Figure 1E**). Collectively, these results indicated that GA could ameliorate BLM-induced pulmonary fibrosis in a dose-dependent manner.

### GA Mitigated Inflammation and Oxidative Stress in BLM-induced Pulmonary Fibrosis

**FIGURE 2:**
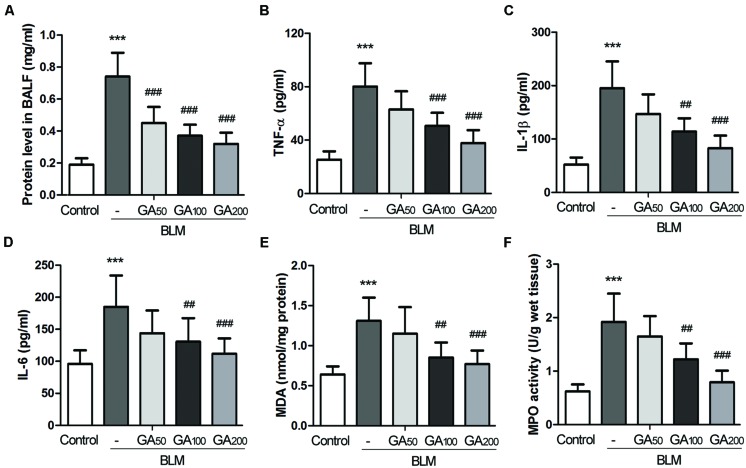
**Glycyrrhizic acid reduced BLM-induced elevation of inflammatory cytokines and oxidative stress in the lungs.** BALF was collected after the rats were sacrificed. **(A)** The total protein level in the BALF was determined by the BCA assay kit. **(B–D)** The levels of TNF-α, IL-1β and IL-6 in the BALF were assayed by ELISA. **(E)** The MDA content and **(F)** the MPO activity in the lungs were examined by the respective kits. The results are presented as the mean ± standard deviation (*n* = 8 per group). Compared with the control group, ***p < 0.001; compared with the BLM group, ^##^*p* < 0.01,^###^*p* < 0.001.

To determine the effect of GA on BLM-induced pulmonary inflammatory responses in rats, the total and differential counts of leukocytes in BALF were firstly determined. Compared with the control rats, a significant influx of inflammatory cells was observed in the BALF from the rats with BLM-induced pulmonary fibrosis, and the numbers of neutrophils, macrophages and lymphocytes were all increased (**Table 1**). Following 28-days GA treatment, BLM-induced increases of inflammatory cell counts were markedly reduced, and such inhibitory effect was dose dependent. In addition, BLM induction resulted in elevated levels of total protein and various inflammatory cytokines in the BALF such as TNF-α, IL-1β and IL-6, which were all reduced by GA treatment in a dose dependent manner (**Figures 2A–D**).

**Table 1:** Effect of GA on BLM-induced changes in total and differential cell counts in the bronchoalveolar lavage fluid of rats.

Group	Total cells (×10^5^)	Neutrophils (×10^4^)	Macrophages (×10^5^)	Lymphocytes (×10^4^)
Control	2.55 ± 0.72	4.93 ± 1.66	1.69 ± 0.52	3.69 ± 1.62
BLM	11.0 ± 1.74***	12.2 ± 3.63***	8.84 ± 1.46***	9.82 ± 2.76***
BLM+GA_50_	9.05 ± 2.38	9.4 ± 3.36	7.27 ± 1.87	8.4 ± 3.04
BLM+GA_100_	6.66 ± 1.91^##^	8.52 ± 2.32	5.16 ± 1.57^###^	6.5 ± 1.97
BLM+GA_200_	5.42 ± 1.13^###^	7.13 ± 1.81^#^	4.16 ± 0.94^###^	5.45 ± 1.06^#^

Data are expressed as the mean ± standard deviation (*n* = 8 per group); Compared with the control group, ***p < 0.001. Compared with the BLM group, ^#^*p* < 0.05, ^##^*p* < 0.01, ^###^*p* < 0.001. GA, glycyrrhizic acid; BLM, bleomycin.

Compared with the control rats, a significant elevation in MDA content, an indicator of lipid peroxidation (Torun et al., 2009), was observed in the lung tissues of the rats exposed to BLM, whereas GA treatment at a dose of 100 and 200 mg/kg bw/d significantly inhibited BLM-induced elevation of MDA in the lungs (**Figure 2E**). Meanwhile, the level of MPO, a marker of neutrophil influx and oxidative stress (Pitanga et al., 2014), was increased in BLM-treated lungs, and such increase was remarkably attenuated by GA treatment in a dose-dependent manner (**Figure 2F**). Thus, these results suggest that GA could combat oxidative stress in BLM-induced pulmonary fibrosis.

### GA Inhibited EMT and Activation of TGF-β Signaling Pathway

**FIGURE 3:**
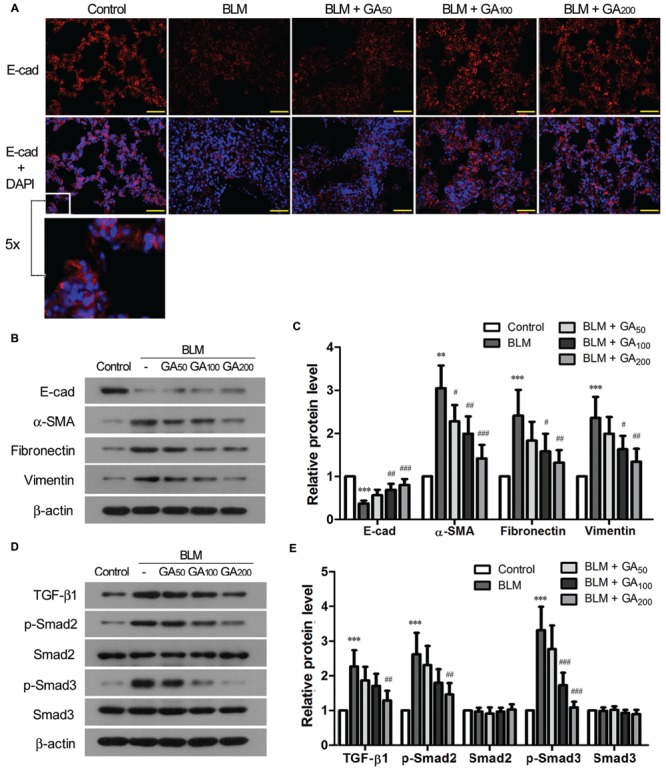
**Glycyrrhizic acid inhibited EMT and activation of TGF-β signaling in BLM-induced pulmonary fibrosis. (A)** Immunofluorescence staining of E-cadherin (E-cad) in the lungs. The sections were photographed at 400× magnification (scale bars = 50 μm). A small window is enlarged by five times to present the subcellular location of E-cad. **(B)** Immunoblotting for the epithelial marker (E-cad), myofibroblasts maker (α-SMA), and ECM molecules (Fibronectin and Vimenin) in the lung tissues. **(C)** Densitometric analysis of proteins of interest in the immunoblots using β-actin as the internal reference. Values are expressed as the mean ± standard deviation (*n* = 8 per group). Compared with the Control group, **p < 0.01, ***p < 0.001; Compared with the BLM group, ^#^*p* < 0.05, ^##^*p* < 0.01, ^###^*p* < 0.001. **(D)** Immunoblotting for TGF-β1 and its downstream signaling molecules (p-Smad2 and p-Smad3) in the lung tissues. **(E)** Densitometric analysis of target proteins in **(D)** using β-actin as the internal reference. Values are expressed as the mean ± standard deviation (*n* = 8 per group). Compared with the Control group, ***p < 0.001; Compared with the BLM group, ^##^*p* < 0.01, ^###^*p* < 0.001.

Epithelial-mesenchymal transition is one of the well-recognized sources of myofibroblasts in pulmonary fibrosis (Willis and Borok, 2007). The epithelial marker E-cadherin, which was expressed in the alveolar septa of normal lungs, was markedly down-regulated in BLM-induced pulmonary fibrosis, while the expression of E-cadherin was resumed in the rats that received medium to high dose of GA (**Figure 3A**). Western blot analysis revealed that α-SMA was upregulated in BLM-treated lungs along with the downregulation of E-cadherin, suggesting an enhanced EMT process in BLM-induced pulmonary fibrosis (**Figures 3B,C**). In contrast, GA treatment inhibited BLM-induced EMT in the lungs by increasing the expression of E-cadherin and simultaneously suppressing the expression of α-SMA after BLM induction. Moreover, GA treatment decreased BLM-induced upregulation of ECM molecules such as Fibronectin and Vimentin, which are normally secreted by myofibroblasts during fibrosis (**Figures 3B,C**).

Transforming growth factor-β signaling is a well-known signaling pathway that plays a critical role in EMT during pulmonary fibrosis (Willis and Borok, 2007). Hence, we examined the activation status of TGF-β signaling pathway in BLM-induced pulmonary fibrosis with and without GA treatment. Compared with the control lungs, the expression of TGF-β1 was significantly upregulated in BLM-induced lungs, accompanied by enhanced phosphorylation of the signaling molecules downstream of TGF-β1, including Smad2 and Smad3 (**Figures 3D,E**). GA treatment of medium and high doses, on the other hand, suppressed BLM-induced upregulation of TGF-β1 as well as decreased the elevation of p-Smad2 and p-Smad3. These results suggest an inhibitory effect of GA on BLM-induced activation of TGF-β signaling in the lungs.

### GA Suppressed Growth and Induced Apoptosis of Fibroblasts

**FIGURE 4:**
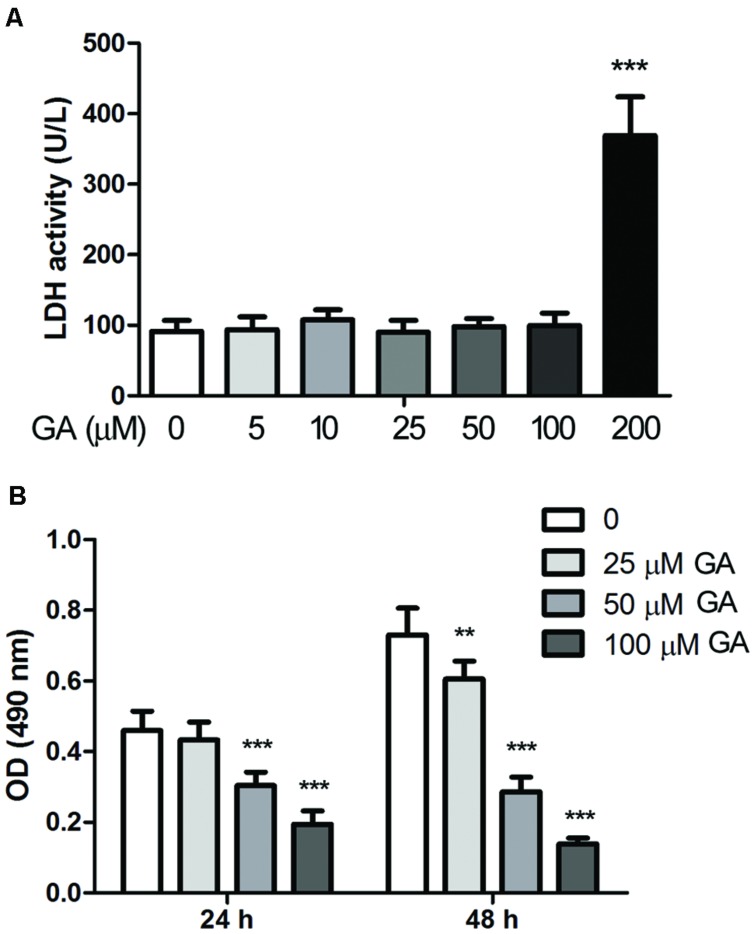
**Glycyrrhizic acid suppressed proliferation of 3T6 fibroblast cells. (A)** Murine fibroblast cell line 3T6 was treated with various concentrations of GA for 24 h, and the cytotoxicity of GA was detected by the LDH activity in the conditioned culture medium. **(B)** The proliferation of 3T6 cells was assessed by MTT assay after 24 and 48 h GA treatment. The results are presented as the mean ± standard deviation of three independent experiments. Compared with the untreated cells, **p < 0.01, ***p < 0.001.

**FIGURE 5:**
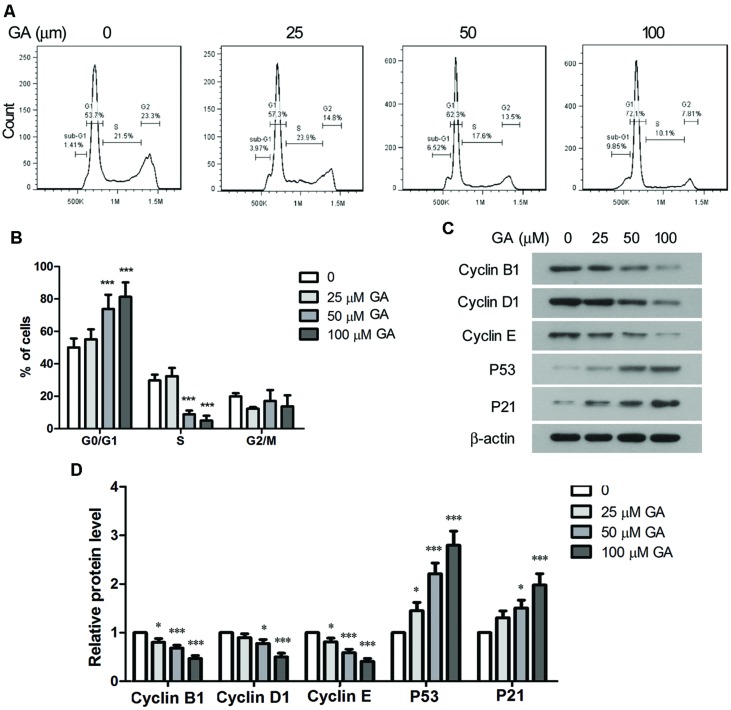
**Glycyrrhizic acid induced cell cycle arrest of 3T6 cells. (A)** 3T6 cells were treated with the indicated concentration of GA for 24 h. The cells were fixed and stained with PI, followed by flow cytometric analysis. **(B)** The flow cytometric results from three independent experiments were statistically analyzed. **(C)** Western blot analysis of the key cell cycle regulatory proteins in 3T6 cells after 24 h GA treatment. **(D)** Densitometric analysis of proteins of interest in the immunoblots using β-actin as the internal reference. This figure shows the representative images from three independent experiments, and the data are expressed as the mean ± standard deviation. Compared with the untreated cells, *p < 0.05, ***p < 0.001.

Proliferation of fibroblasts takes place at the initial stage of tissue repair in response to injury, and the tightly regulated growth and apoptosis of fibroblasts are critical to restore normal tissue architecture (Lorena et al., 2002). The effect of GA on the proliferation and apoptosis of fibroblasts was investigated *in vitro* by employing a murine fibroblast cell line 3T6. The cytotoxicity of GA on 3T6 cells were examined by incubating the cells with different concentrations of GA ranging from 5 to 200 μM for 24 h, and the LDH activity assay indicated that GA of as high as 100 μM was non-cytotoxic to 3T6 cells (**Figure 4A**). Later on, the effect of GA on the proliferation of 3T6 cells was assessed by MTT assay. It was noted that GA treatment at the doses of 50 and 100 μM led to significant reduction of viable cell numbers after 24 and 48 h incubation as compared to the untreated cells, whereas 25 μM GA displayed the inhibitory effect on cell proliferation only at 48 h (**Figure 4B**). We further examined the cell cycle of 3T6 cells after 24 h treatment with GA. The results revealed that GA of 50 and 100 μM markedly increased the numbers of cells at G_0_/G_1_ phase and reduced the numbers of cells at S phase (**Figures 5A,B**), indicating that the cell cycle was slowed down by GA treatment. Consistently, GA of 50 and 100 μM led to downregulated expression of a number of cell cycle regulatory proteins such as Cyclin B1, Cyclin D1 and Cyclin E, and upregulated the G1 check-point proteins including P53 and P21 (**Figures 5C,D**). In addition, GA inhibited cell cycle and altered the expression of the cell cycle players in a dose-dependent manner.

**FIGURE 6:**
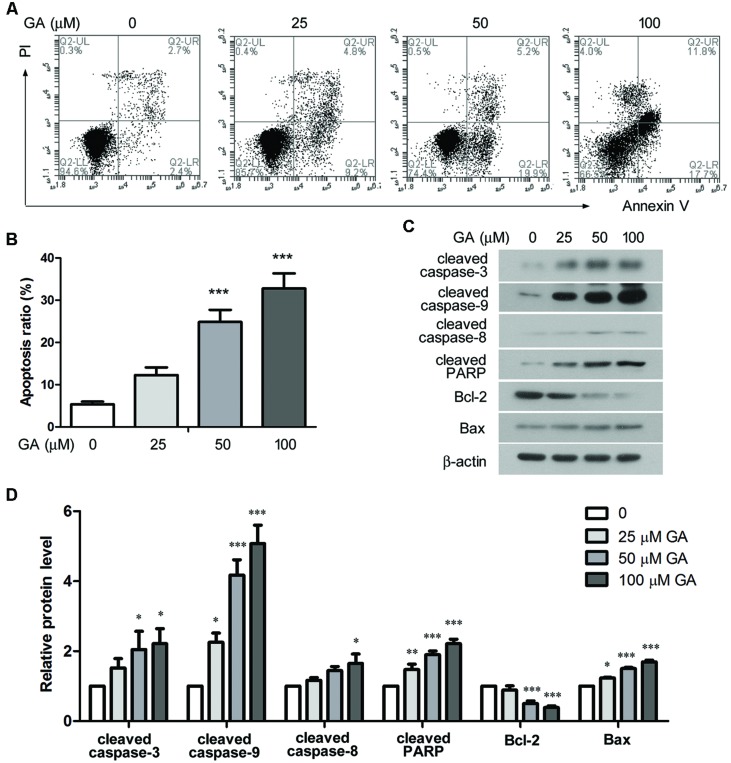
**Glycyrrhizic acid promoted apoptosis of 3T6 cell. (A)** Following 24 h GA treatment, 3T6 cells were double-stained with Annexin-V-FITC and PI, and then analyzed by flow cytometry. **(B)** The ratio of apoptotic cells (Annexin V+ PI- and Annexin V+ PI+) was statistically analyzed. **(C)** Western blot analysis of several key apoptosis regulatory proteins in 3T6 cells after 24 h GA treatment. **(D)** Densitometric analysis of proteins of interest in **(C)** using β-actin as the internal reference. **(A,C)** Shows the representative images from three independent experiments. The values are expressed as the mean ± standard deviation. Compared with the untreated cells, *p < 0.05, **p < 0.01, ***p < 0.001.

Cell apoptosis was analyzed by flow cytometry after 24 h treatment of GA. The ratio of the cells that were stained as Annexin V+ PI- and Annexin V+ PI+, representing early and late apoptotic cells, was statistically analyzed, and the results indicated that GA enhanced apoptosis of 3T6 cells in a dose-dependent manner (**Figures 6A,B**). Further, Western blot results showed that the levels of cleaved caspase-3, cleaved caspase-9 and cleaved PARP, the executors of apoptotic events, were markedly increased by GA, while the level of cleaved caspase-9 was elevated to a less extent (**Figures 6C,D**). Moreover, GA treatment resulted in downregulation of the anti-apoptotic protein Bcl-2 and upregulation of the pro-apoptotic protein Bax (**Figures 6C,D**). Thus, the altered levels of the critical apoptotic players favored the apoptotic phenotype of 3T6 cells after GA treatment.

### GA Inhibited Migration and Invasion of 3T6 Cells

**FIGURE 7:**
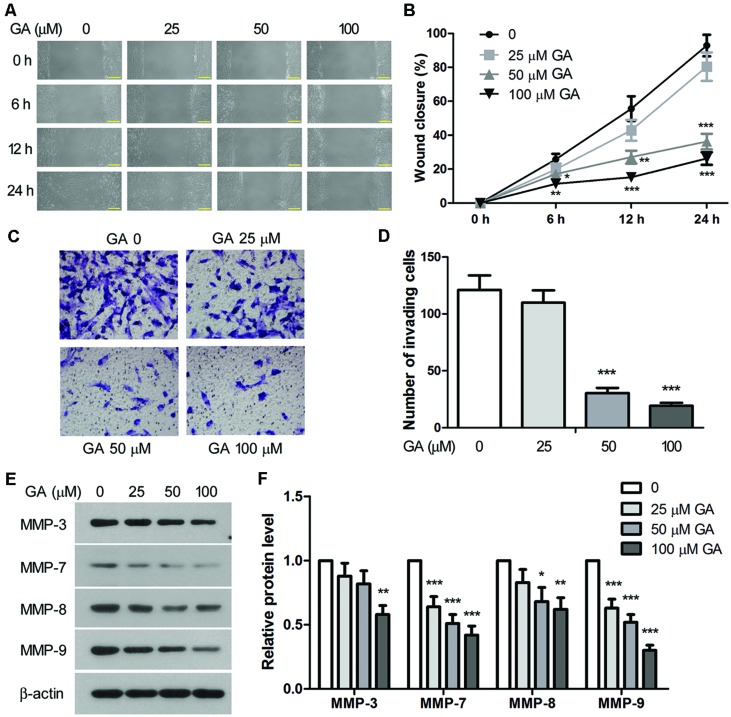
**Glycyrrhizic acid inhibited migration and invasion of 3T6 cells. (A)** The effect of GA on cell migration was assessed by scratch wound assay. The wound closure was photographed at post-scratching 6, 12, and 24 h. **(B)** The wound closure rate, representing the migration rate, was measured. **(C)** Transwell assay was performed to evaluate the effect of GA on the invasiveness of 3T6 cells. The cells that migrated through the membrane were stained and photographed. **(D)** The number of invading cells in the Transwell assay was statistically analyzed. **(E)** Immunoblotting for the MMP family proteins in 3T6 cells after treatment with various concentrations of GA. **(F)** Densitometric analysis of MMPs in the immunoblots using β-actin as the internal reference. **(A,C,E)** present the representative images from three independent experiments, and the values are expressed as the mean ± standard deviation. Compared with the untreated cells, *p < 0.05, **p < 0.01, ***p < 0.001.

*In vitro* scratch wound assay and Transwell assay were performed to assess the migration and invasion of 3T6 cells after GA treatment. As shown in **Figures 7A,B**, the migration rate of 3T6 cells was significantly reduced by GA at 50 and 100 μM since 6 h of the assay. Notably, the invasiveness of 3T6 cells was strongly inhibited during 24 h incubation with 50 or 100 μM GA (**Figures 7C,D**). Thus, these results demonstrated that GA at the concentration of 50 or 100 μM significantly suppressed migration and invasion of 3T6 cells.

Matrix metalloproteinases (MMPs) are endopeptidases enzymes capable of degrading ECM components during cell invasion, and they have been implicated in the pathology of pulmonary fibrosis (Craig et al., 2015). Here, we show that treatment with GA led to dose-dependent downregulation of MMP-3, MMP-7, MMP-8, and MMP-9 proteins in 3T6 cells (**Figures 7E,F**), suggesting that GA may inhibit cell invasion by interfering with the expression of these MMPs.

## Discussion

Intratracheal administration of BLM is one of the most extensively used experimental models in the studies of pulmonary fibrosis at present. BLM is known to stimulate inflammatory responses in the lung tissues with increased infiltration of leukocytes such as macrophages, granulocytes, and lymphocytes. These inflammatory cells stimulate the proliferation of fibroblasts and the activation of myofibroblasts which secrete ECM components into the alveolar interstitial space, gradually resulting in pulmonary fibrosis (Mouratis and Aidinis, 2011). In our study, intratracheal administration of BLM led to the typical phenotypes of BLM-induced pulmonary fibrosis, including marked infiltration of inflammatory cells into the lung tissues, elevation of inflammatory cytokines, excessive proliferation of fibroblasts, massive production of collagen fibers, and deposition of ECM molecules in the lungs. These pathological alterations were all significantly alleviated by intraperitoneal injection of GA at 100 and 200 mg/kg bw/d for 28 days, and the therapeutic effect of GA on pulmonary fibrosis was dose dependent. Hence, our results suggest a potential value of GA in the treatment of IPF.

Idiopathic pulmonary fibrosis is a heterogeneous pulmonary disorder of undefined etiology. Dysregulated fibroproliferation in response to alveolar epithelial injury has drawn extensive attention in recent years, and current understanding of the pathogenesis of IPF is shifting from the conventional concept of chronic inflammation toward dysregulated fibroproliferation (Ryu et al., 2014). In this study, we demonstrated that GA inhibited the proliferation of 3T6 fibroblasts by upregulating the cell cycle checkpoint proteins and downregulating several Cyclins. In addition, GA induced apoptosis of 3T6 cells via modulating the levels of a number of key apoptosis regulatory proteins. Particularly, GA treatment augmented the cleavage of caspase-3 and caspase-9 to a much greater extent as compared to GA-induced cleavage of caspase-8, suggesting that GA-stimulated apoptosis is predominantly mediated via the intrinsic apoptotic pathway (Bayir and Kagan, 2008). Apoptosis of fibroblasts is critical at the end of the healing process for restoration of normal tissue architecture (Desmouliere et al., 1995). Thus, it is likely that GA treatment inhibited proliferation and induced apoptosis of fibroblasts during the development of pulmonary fibrosis in BLM-treated rats, thus attenuating BLM-induced fibroproliferation, such as to delay the pathogenesis of pulmonary fibrosis.

TGF-β1 has been implicated in a broad spectrum of activities in the pathogenesis of pulmonary fibrosis such as pulmonary inflammation (Bergeron et al., 2003), differentiation of fibroblasts into active myofibroblasts and inhibition of fibroblast apoptosis (Lee et al., 2006), EMT (Willis and Borok, 2007), as well as synthesis and deposition of collagen and ECM molecules by myofibroblasts (Ignotz and Massague, 1986). Previous studies have demonstrated that TGF-β is the major inducer of EMT in fibrosis via Smad-dependent pathways (Li et al., 2003). In the present study, intratracheal administration of BLM led to increased expression of α-SMA in the lungs, implying an increased number of myofibroblasts, which may be derived from fibroblast transdifferentiation or EMT. GA treatment inhibited BLM-induced upregulation of α-SMA and also reduced BLM-induced upregulation of TGF-β1 and phosphorylation of Smad2/3, suggesting that GA may mitigate BLM-induced pulmonary fibrosis by inhibiting TGF-β signaling pathway.

Previous studies have proposed a therapeutic potential of GA against liver fibrosis (Guo et al., 2013; Liang et al., 2015). Our study demonstrates, for the first time, that GA can ameliorate pulmonary fibrosis in a rat model of BLM-induced pulmonary fibrosis by reducing the infiltration of inflammatory cells, inhibiting the production of inflammatory cytokines, decreasing oxidative stress and suppressing fibroblast proliferation. Since GA is known to possess anti-inflammatory, anti-oxidant and anti-apoptotic properties (Guo et al., 2013; Ming and Yin, 2013; Liang et al., 2015), the therapeutic effect of GA against BLM-induced pulmonary fibrosis might be attributed to these diverse pharmaceutical properties of GA. A previous work by Moro et al. proposed Smad3-mediated transcription of collagen I as the molecular mechanism for the anti-fibrotic effect of GA (Moro et al., 2008). Here, we showed that GA did not only interfere with the activation of Smad3, but also affected the phosphorylation of Smad2 and the expression of TGF-β1. Therefore, our results suggest that GA may exert the anti-fibrotic effect by suppressing the activation of TGF-β signaling pathway, probably through the modulation of TGF-β1 expression.

The levels of most MMP family proteins are elevated in IPF, and several MMPs have been demonstrated to promote pulmonary fibrosis in animal models mainly by upregulating the pro-fibrotic mediators, downregulating the anti-fibrotic mediators, and enhancing cell migration (Craig et al., 2015). MMP-3 and MMP-7, in particular, have been shown to promote the EMT process during the development of pulmonary fibrosis (Zuo et al., 2002; Yamashita et al., 2011). In this study, GA dose-dependently reduced the expression of MMP-3, -7, -8, and -9 in 3T6 cells, suggesting that GA-induced downregulation of MMPs may contribute to the anti-fibrotic action of GA.

In the past few decades, animal models of BLM-induced pulmonary fibrosis have provided important insights in the understanding of IPF pathogenesis. Notably, there are emerging concerns about this model regarding the severe adverse effect of lung injury, which leads to IPF-like phenotypes but only partially recapitulate the disease. Moreover, although a great number of novel compounds were demonstrated to be promising based on this model, most of them fail to show efficacy in clinical trials (Della Latta et al., 2015). Hence, in future studies, alternative models of pulmonary fibrosis may be recruited to confirm the pharmaceutical property of GA, either by chemical induction or using transgenic animals (Wilberding et al., 2001; Moore and Hogaboam, 2008).

## Conclusion

Our study demonstrated that GA treatment significantly alleviated BLM-induced pulmonary fibrosis in rats, providing the preliminary evidence for the potential therapeutic value of GA for IPF.

## Author Contributions

HL and TW conceived the study. LG and HT carried out the animal model construction and the treatment. LG, HT, HH, and JL performed the examinations of the animal samples. JM and HJ conducted the cell line work. HL and TW reviewed the data and drafted the manuscript.

## Conflict of Interest Statement

The authors declare that the research was conducted in the absence of any commercial or financial relationships that could be construed as a potential conflict of interest.
